# Free Energy Profile for the Complete Transport of Nonpolar Molecules through a Carbon Nanotube

**DOI:** 10.3390/ijms241914565

**Published:** 2023-09-26

**Authors:** Changsun Eun

**Affiliations:** Department of Chemistry, Hankuk University of Foreign Studies, Yongin 17035, Republic of Korea; ceun@hufs.ac.kr

**Keywords:** molecular dynamics simulation, molecular transport, nanochannel, potential of mean force, PMF, carbon nanotube, CNT, free energy, graphene

## Abstract

Gas molecules or weakly interacting molecules are commonly observed to diffuse through and fill space. Therefore, when the molecules initially confined in one compartment are allowed to move through a channel into another empty compartment, we expect that some molecules will be transported into the initially empty compartment. In this work, we thermodynamically analyze this transport process using a simple model consisting of graphene plates, a carbon nanotube (CNT), and nonpolar molecules that are weakly interacting with each other. Specifically, we calculate the free energy change, or the potential of mean force (PMF), as the molecules are transported from one compartment to another compartment. The PMF profile clearly exhibits a global minimum, or a free energy well, at the state wherein the molecules are evenly distributed over the two compartments. To better understand the thermodynamic origin of the well, we calculate the energetic and entropic contributions to the formation of the well, and we show that the entropic change is responsible for it and is the driving force for transport. Our work not only enables a fundamental understanding of the thermodynamic nature of the transport of weakly interacting molecules with molecular details, but also provides a method for calculating the free energy change during transport between two separate spaces connected by a nanochannel.

## 1. Introduction

Molecular transport occurs everywhere. However, the driving forces for molecular transport differ from one transport system to another. For example, ion transport through ion channels in cells is driven by an electric potential [1], water transport in plant roots is governed by osmosis [2], and water transport in reverse osmosis membranes relies on a pressure gradient [3,4,5]. Nonetheless, the quantitative analysis of the thermodynamic details of molecular transport in such systems is limited due to their inherent complexity.

To overcome this analytical challenge and take a preliminary step toward understanding more intricate and realistic systems, we recently developed a simple model. This model consists of two compartments connected by a carbon nanotube (CNT), which simplifies analysis due to its well-defined simple geometry and the presence of only one type of transported molecule [6,7,8]. Using this model, we calculated the free energy change, or the potential of mean force (PMF), for the complete transport of water molecules from one compartment to the other [8].

In our previous work with water [8], the PMF showed that, in complete transport, the transport of water from one compartment (Compartment 1) to the CNT is spontaneous, but further transport to the other compartment (Compartment 2) is not spontaneous. In fact, the PMF profile exhibits a barrier in the middle, indicating that the state in which half of the water molecules are transported into the other compartment is thermodynamically unstable. This barrier arises due to the strong interactions between water molecules, which are manifested by hydrogen bonds.

Specifically, during the initial phase of transport, water molecules initially in Compartment 1 split into two groups across Compartment 1 and Compartment 2 as they pass through the CNT. This process leads to the loss of hydrogen bonds and an increase in free energy. However, in the subsequent phase, the two groups merge within Compartment 2, resulting in a gain in hydrogen bonds and a decrease in free energy. Consequently, throughout the entire process, free energy first increases and then decreases, giving rise to the appearance of a barrier. Further analysis of the thermodynamics of water transport indicates that the energetic contribution to the PMF change outweighs the entropic contribution, with the interactions between water molecules being primarily responsible for this energetic contribution.

Instead of water molecules, we now consider cases wherein the transported molecules exhibit weak interactions with each other, resulting in molecular interactions that are significantly weaker than those of water molecules. In this work, we explore these cases using a slightly modified model based on the one employed for water transport. We calculated the PMF for various scenarios involving different numbers of transported molecules and estimated the amount of free energy required to transport all molecules between two compartments, which is represented by the PMF well depth. Furthermore, we discuss how the PMF for weakly interacting molecules generally differs from that for water.

This paper is organized as follows. In Section 2, we describe the details of our model and explain how we can calculate the PMF. In Section 3, we present our PMF results and discuss their physical significance. In Section 4, we summarize our findings and provide some implications of our work.

## 2. Materials and Methods

### 2.1. Computational Models

In this work, the primary objective is to calculate the change in free energy resulting from the transport of weakly interacting molecules. To achieve this, we developed a suitable model and utilized an appropriate calculation method for the PMF. To ensure the model’s appropriateness, we aimed to keep it as simple as possible to facilitate a clear understanding of the underlying process. Simultaneously, we retained the fundamental aspects of molecule transport. For such a model, we essentially adopted an approach that has demonstrated its utility in our prior investigations of osmosis-induced molecular transport [6,7] and the PMF calculation for the complete transport of water molecules between compartments [8].

**Figure 1:**
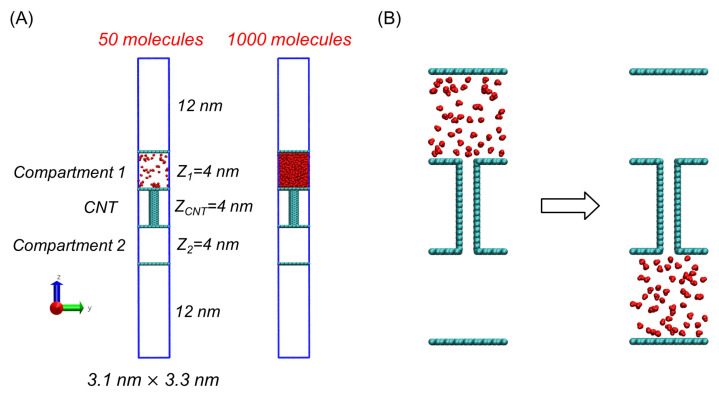
Transport of nonpolar molecules from one compartment (Compartment 1) to another empty compartment (Compartment 2) through a CNT. (**A**) Configurations of the systems involving 50 (**left**) and 1000 (**right**) transported molecules, showing all molecules located in Compartment 1. The two systems are identical except for the number of transported molecules. Blue lines represent the periodic boundaries of the systems. (**B**) Initial (**left**) and final (**right**) states of molecular transport involving 50 molecules. These states are considered for the PMF calculation.

In the model, we constructed two separate spaces connected by a CNT, through which molecules are transported from one space to the other. Each space, or compartment, is made of two graphene plates (see Figure 1A). In Figure 1A, the topmost and bottommost plates were essential for blocking the passage of molecules between the compartments through the simulation boundaries, thus making passage through the CNT the only possible way to transport molecules between the compartments. For the CNT itself, we used a (6, 6) CNT with a length of 4 nm, whose width is so small that only one molecule can pass through it at a time. To prepare weakly interacting molecules, we employed the water model used in our previous works [6,7,8] and simply removed the electric charges, thereby completely eliminating electrostatic interactions responsible for strong interactions or hydrogen bonds [6]. Furthermore, to weaken the interactions even more than those lacking electrostatic interactions, we considered reducing the Lennard–Jones (LJ) interaction strength. However, based on findings from our prior study using this charge-removed water model [6], we concluded that the removal of electrostatic interactions alone is sufficient to induce gas-like behavior. As a result, we utilized this charge-removed water model consistently throughout this study.

In fact, instead of using this artificial, charge-removed water molecule, it could be more compelling to employ a realistic nonpolar molecule that can be compared to experimental results. However, using this approach would necessitate the development of an accurate force field for the chosen realistic molecule [9], and it may also require the use of ab initio molecular dynamics simulation methods [10,11,12]. Importantly, given the focus of our research on the general shape of the PMF for molecular transport, delving into these complexities falls outside the scope of our current study. Our model molecule, which exhibits gas-like properties [6], adequately serves our research objectives.

Moreover, an additional advantage of utilizing this charge-removed water molecule, or weakly interacting nonpolar molecule, is that it provides a clear understanding of the exclusive influence of the strength of interaction between transported molecules on molecular transport. This clarity emerges through a comparison with the model featuring strongly interacting water molecules, where all other factors precisely match those of water molecules. In this work, we examined eight cases involving 50, 100, 200, 400, 600, 800, 884, and 1000 nonpolar molecules, similar to the cases involving water molecules [8].

### 2.2. Molecular Dynamics Simulations

In this study, to calculate the free energy differences between the observable states in molecular transport, we sampled the states in the transport process. For this purpose, we used a molecular dynamics (MD) simulation method with umbrella sampling. To run MD simulations, we used the AMBER force field [13,14] for the CNT (see Table 1), and for nonpolar molecules, we employed the TIP3P water model [15], which was modified by neutralizing all electric charges (see Table 2). For the graphene plates, we utilized a slightly modified version of the AMBER force field that we previously employed in our studies involving water molecules [7,8]. Specifically, the LJ parameters of the carbon atoms in the graphene plates are ε=0.03598 kJ/mol and σ=0.3400 nm (see Table 1). Note that the LJ parameters (ε and σ) are defined through the LJ potential V given by $V\left(r\right)=4\varepsilon \left({\left(\frac{\sigma }{r}\right)}^{12}-{\left(\frac{\sigma }{r}\right)}^{6}\right)$, where r is the distance between atoms. For the LJ potential between different types of atoms, unless otherwise specified, the LJ parameters were computed using the Lorentz–Berthelot rule [16]. The cutoff distance for LJ interactions was 1.4 nm. Throughout the MD simulations, periodic boundary conditions (PBCs) were applied in all xyz directions.

The transport system was centered within a simulation box with a z length of 32 nm, which is significantly longer than the system’s z length of 12 nm (see Figure 1A). The rationale for using this expanded simulation box was to minimize interactions between the system and its periodic images along the z-axis due to the PBCs.

To perform MD simulations, we used the GROMACS package version 2020.6 [17]. During the simulations, the integration time step was 2.0 fs, and we immobilized the positions of the CNT and the compartments using the freeze group option provided by the package [16]. All simulations were performed under NVT conditions. To maintain a constant temperature (T) of 300 K, we employed the modified Berendsen algorithm named the V-rescale thermostat [18] with a coupling constant of 0.1 ps, as we did in the previous study of water transport [8]. For visualization and analysis of the simulation results, we utilized the VMD version 1.9.4 [19] (http://www.ks.uiuc.edu/Research/vmd/; accessed on 29 January 2019; University of Illinois at Urbana-Champaign, Urbana, IL, USA) and xmgrace version 5.1.25 (http://plasma-gate.weizmann.ac.il/Grace/; accessed on 27 October 2017) programs.

### 2.3. Potential of Mean Force

In this work, our focus was on examining the intricate details of the change in free energy that occurs as molecules traverse from one compartment to another through a CNT. Consequently, Figure 1B illustrates the desired initial and final states. Between them, there are intermediate states. To specify these states for the PMF calculation, we considered the number of molecules in Compartment 1 ($N_{1}$), which was used as the coordinate of the PMF. That is, when $N_{1}$ is equal to N, the total number of molecules, the corresponding state is that all molecules are in Compartment 1. Similarly, when $N_{1}$ is N/2, half of the molecules are in Compartment 1, and the other half are in Compartment 2 and the CNT.

To sufficiently sample these states, we carried out MD simulations with the initial configurations representing the desired states to be sampled. To prepare the initial configurations, we used the configurations of water molecules used in our previous work [8]. The key in the sampling was that, during the simulation, the sampled states should be around the desired states with a specific value of $N_{1}$. This was made possible by applying umbrella potentials to the system in the desired states. Specifically, to keep $N_{1}$ fixed, we applied an umbrella potential with a force constant of 3000 kJ/mol to a specific molecule in the CNT. In this way, the molecule in the CNT was constrained in space and consequently blocked the molecular transport, which means that $N_{1}$ was almost constant.

**Figure 2:**
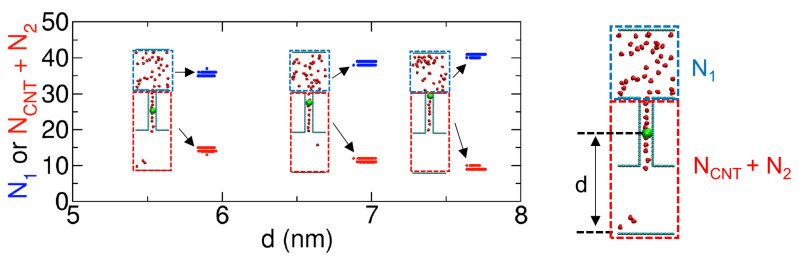
Changes in the numbers of molecules in Compartment 1 ($N_{1}$; counted in the blue dashed boxes) and the remaining space ($N_{CNT}+N_{2}$; counted in the red dashed boxes) for three 1 ns sampling simulations with an umbrella potential for the PMF calculation, involving 50 molecules. The umbrella potential was applied to one molecule in green, constraining the distance (d) from the bottom plate in Compartment 2. The PMF calculated from these samplings is shown in Set 2 in Figure 3A. Note that $N_{1}+N_{CNT}+N_{2}=50$, and the green molecules in the three cases are the same molecule.

For example, in each of the three sampling simulations shown in Figure 2, we observe that, due to the strong constraint on a specific molecule (green) within the CNT, $N_{1}$ (blue circles) remains nearly constant. Consequently, the total number of molecules in the remaining space, $N_{CNT}+N_{2}$ (red circles), also remains almost constant. We also see that, as we move the molecule (green) within the CNT and then apply the constraint force to it, we can sample states with different values of $N_{1}$. Here, it is worth noting that the three snapshots in the inset of Figure 2 can be considered as images capturing the movement of the green-colored molecule, whether it is moving from above to below or from below to above. In this context, the constraint force serves to hold the green molecule, ensuring the right moment. This umbrella sampling was successfully performed in our previous work [8] for the PMF calculation of water transport. For more details, one can refer to our previous work [8].

One notable aspect concerning the coordinate $N_{1}$ of the PMF is that we have introduced negative values of $N_{1}$ [8], in addition to the naturally defined positive values. To be more precise, a negative value indicates how many molecules are required to completely fill the CNT inner space while Compartment 1 remains empty or to become the state with $N_{1}=0$. Notably, in our model, there are approximately 11 to 12 molecules in the fully occupied CNT space. Thus, by definition, the state with $N_{1}=-1$ means that, from the CNT fully occupied state with $N_{1}=0$, one molecule is transported from the CNT space to Compartment 2, while there is still no molecule in Compartment 1. This extended definition of $N_{1}$ was necessary to represent all the states with the single coordinate $N_{1}$ and thus depict all the changes in free energy on a two-dimensional graph wherein free energy is plotted against the $N_{1}$ coordinate.

**Figure 3:**
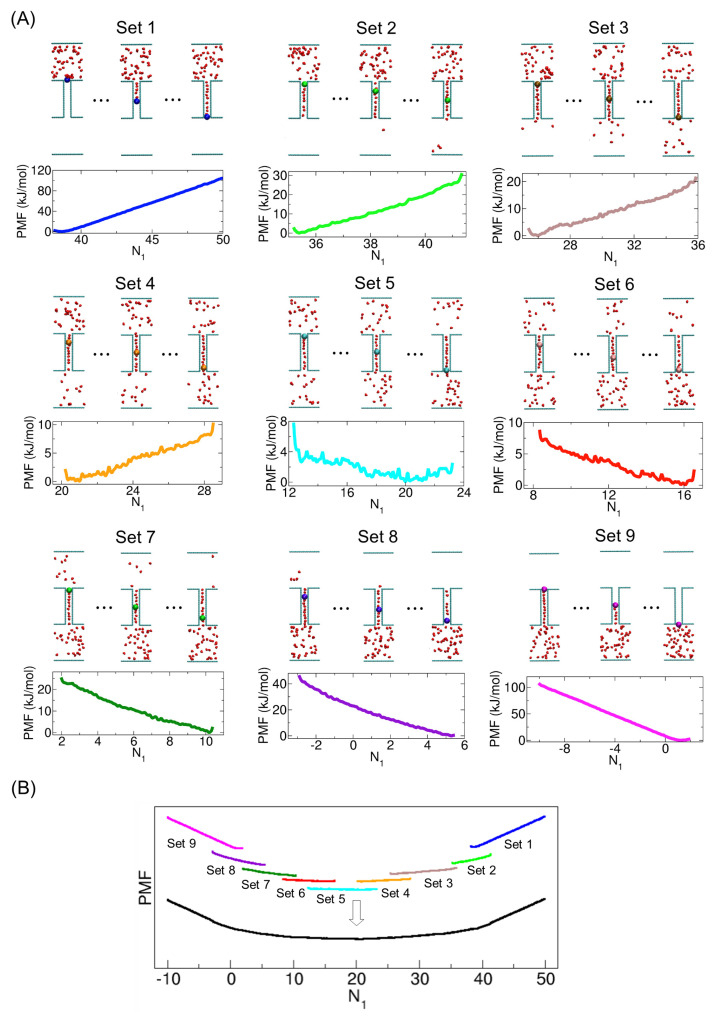
(**A**) Nine sets of umbrella samplings for the PMF calculation of 50 nonpolar molecules. In each window of a sampling set, during the simulation, a specific molecule in the CNT, which is distinguished by its larger size and distinct color from the other molecules in the figures, was constrained in space by an umbrella potential. The representative configurations from some windows of these nine sets are illustrated at the top. The PMF calculated from each sampling set is plotted as a function of the number of molecules in Compartment 1 ($N_{1}$) at the bottom. (**B**) Final merged PMF (black) obtained by combining the nine sets of PMFs.

Regarding the details of our sampling simulations, for the sampling, we divided the complete range of states involved in molecular transport into numerous sets of smaller intervals. For example, for the case of 50 molecules, we considered nine sets (see Figure 3A). That is, in each set, we chose a certain molecule within the CNT to be constrained in space. In each sampling window within every set of simulations, using the initial configuration for the desired state, we carried out a 500 ps NVT simulation for equilibration under the umbrella potential and then another 1 ns NVT simulation for sampling, which was used to obtain the PMF. Note that, since we used 768 windows for all nine sets, the total simulation time was 1.152 μs. For each set, we calculated the PMF using the weighted histogram analysis method (WHAM) [20,21]. After calculating the PMF for each set, we merged all the PMFs into one final PMF by shifting the PMFs in a way that the average PMF values in the overlapped regions were the same (see Figure 3B). Finally, we shifted the merged PMF along the y-axis so that the value of the final PMF at $N_{1}=0$ was set to zero (see Figure 3B) because the state with $N_{1}=0$ is well defined as a reference state for any system, irrespective of N. For more details, one can refer to our previous work [8]. Similarly, for a larger number of molecules from N=100 to N=1000, we used the same method described above but with more sampling sets. For example, to calculate the PMFs for 100 and 1000 molecules, we used 18 and 180 sampling sets, respectively, and they correspond to total simulation times of 2.304 μs and 23.04 μs, respectively.

## 3. Results and Discussion

### 3.1. Potential of Mean Force

**Figure 4:**
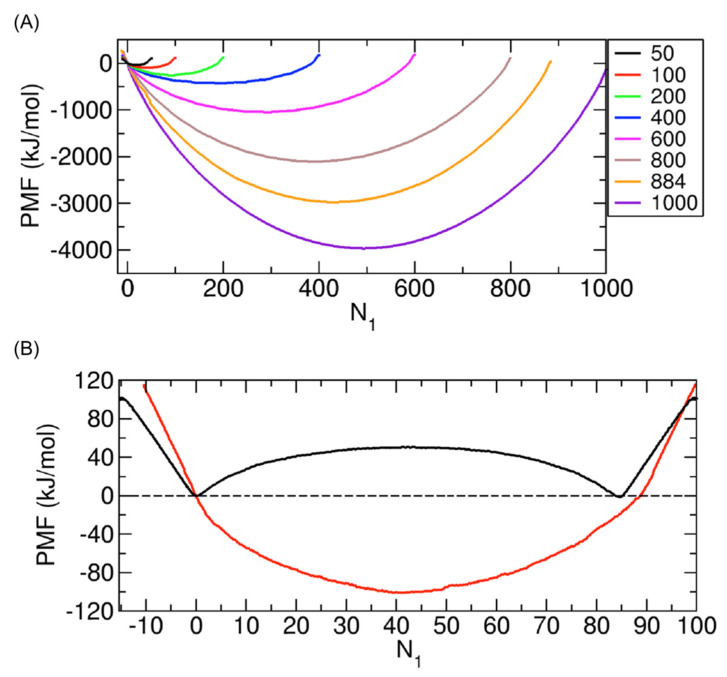
(**A**) PMFs for the cases with 50, 100, 200, 400, 600, 800, 884, and 1000 nonpolar molecules as functions of $N_{1}$. The PMF value at $N_{1}=$ 0 is set to zero. (**B**) PMFs for 100 nonpolar molecules (red) and for 100 water molecules (black). In both cases, there are three distinct regimes, which will be discussed in Figure 5, but their boundaries are not at the same positions of $N_{1}$ but at slightly different positions (approximately, $N_{1}$ = 89 for nonpolar molecules and $N_{1}$ = 84.5 for water molecules), which is due to the different number of molecules needed to completely fill the CNT inner space.

We calculated the PMFs for the cases involving 50, 100, 200, 400, 600, 800, 884, and 1000 nonpolar molecules. The results are presented in Figure 4A, illustrating the PMF profiles. For all cases, we observed distinctive free energy wells at the midpoint of the PMF profiles. These wells exhibit a clear contrast with the free energy barriers observed in the cases involving water molecules [8]; as an example, the PMF with 50 water molecules is demonstrated in Figure 4B. The presence of the well at the midpoint implies that during the complete transport of molecules from Compartment 1 to Compartment 2, nonpolar molecules are spontaneously moved to Compartment 2 until the numbers of molecules in Compartments 1 and 2 are equal or the concentrations in the two compartments are the same. However, the transport of all the remaining molecules in Compartment 1 and the CNT to Compartment 2 is not spontaneous because the process increases the PMF, as shown in Figure 4A. Therefore, to completely transport all molecules, external force is needed for the system to escape from the well to reach the completely transported state. The magnitude of this force is associated with the depth of the well. As shown in Figure 4A, as the total number of transported molecules N increases from 50 to 1000, the depth increases.

**Figure 5:**
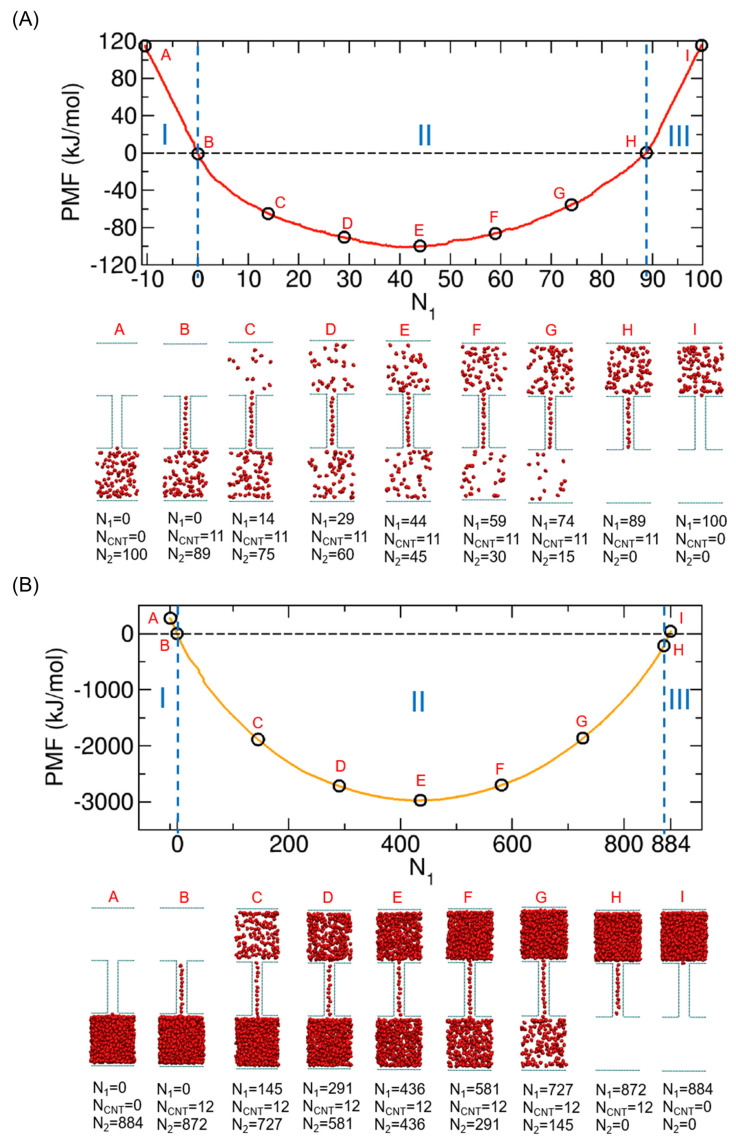
(**A**) (**top**) The PMF of the system with 100 molecules and (**bottom**) the configurations corresponding to the states marked (A–I) in the PMF curve. (**B**) The same figure as the one in (**A**) but with 884 molecules. Here, $N_{1}$, $N_{CNT}$, and $N_{2}$ are the numbers of water molecules in Compartment 1, the CNT and Compartment 2, respectively. Note that $N_{1}+N_{CNT}+N_{2}=100$ in (**A**) and $N_{1}+N_{CNT}+N_{2}=884$ in (**B**). The configurations are representative configurations taken from the umbrella sampling simulations. Three distinct regimes in the PMF are marked as Regimes I, II, and III.

Along with Figure 4A, we also examined the two cases with N=100 and 884 in detail to understand which points in the PMF profile correspond to which transporting states, as we did in our previous study with water molecules [8]. Specifically, we investigated the configurations of the system at specific characteristic points in the PMF (see Figure 5). 

For the 100 molecules in Figure 5A, first, when we examine the PMF change carefully, we see that there are three distinct regimes in the PMFs, although they are not as clearly shown as in the case of water molecules [8] (see Figure 4B). Specifically, in Regimes I and III, the PMF change along $N_{1}$ is linear, but in Regime II, it is not linear. Consequently, there are two boundaries separating these regimes (blue dashed lines in Figure 5A): one is around $N_{1}=N-N_{saturated\;CNT}$ (State H), and the other is around $N_{1}=0$ (State B). Notably, $N_{saturated\;CNT}$ represents the number of molecules in the CNT when it is fully occupied by the molecules, which is approximately 11 (for the 884 molecules in Figure 5B, it is approximately 12). To elaborate further, the boundary state at $N_{1}=N-N_{saturated\;CNT}$ is the state in which Compartment 1 and the CNT are filled with molecules, while Compartment 2 is empty (State H). Similarly, the boundary state at $N_{1}=0$ indicates that Compartment 2 and the CNT are filled with molecules, while Compartment 1 is empty (State B). With these boundaries, Regimes I, II, and III are defined as $N_{1}<0$, $0<N_{1}<N-N_{saturated\;CNT}$, and $N-N_{saturated\;CNT}<N_{1}<N$, respectively.

Based on these three regimes, we now consider the whole transport process from the state in which all the molecules are in Compartment 1 (State I) to the state in which all the molecules are in Compartment 2 (State A), as previously shown in Figure 1B. Consequently, the system is initially in Regime III, and during the transport process in which more molecules are transferred to the CNT, the system is still in Regime III. Thus, we regard the transport process in Regime III as the CNT filling process. However, when the filling process is finished, the state reaches the boundary between Regimes III and II. In Regime II, while the CNT is constantly fully occupied with molecules, the molecules are further transported from the CNT to Compartment 2. After all molecules in Compartment 1 are transported to the CNT and Compartment 2, the state reaches Regime I ($N_{1}<0$). Note that at the boundary with $N_{1}=0$, no more molecules are left in Compartment 1, and thus, the transport process in Regime I has nothing to do with Compartment 1. In Regime I, the molecules remaining in the CNT are finally transported to Compartment 2. Thus, we regard the process in Regime I as the CNT emptying process. 

For the 884 molecules in Figure 5B, we observed the same feature as that in Figure 5A, where there are three distinct regimes, but we also found that the change in Regime II is much more significant than that in Figure 5A. This implies that the increase in the number of molecules mostly affects Regime II and, accordingly, the depth of the well or the minimum PMF.

Additionally, one minor difference we noticed between Figure 5A,B is the average number of molecules when the CNT is filled with them, which is approximately 11 in Figure 5A and 12 in Figure 5B. Since the two systems in Figure 5A,B are identical except for the number of transported molecules, the difference can be attributed to the different numbers of molecules. It appears that the more transported molecules there are in the system, slightly more molecules occupy the CNT inner space. The underlying physical mechanism behind this observation could be a topic of future research.

### 3.2. Well Depth of the Potential of Mean Force

**Figure 6:**
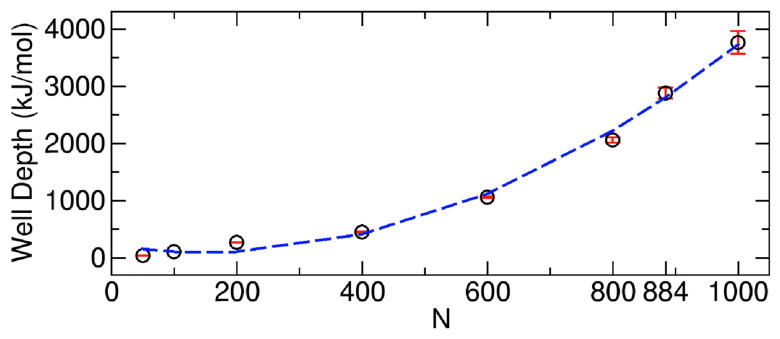
Well depth of the PMFs as a function of the total number of molecules N to be transported. The dashed line is the fitting of the quadratic function to the data. The fitted function is well depth = $0.005033N^{2}-1.521N+208.56$, and the correlation coefficient is 0.997. The well depth was measured as an average of the two differences between the PMF value at N=0 and the minimum of PMF and between the value at $N_{1}=N-N_{saturated\;CNT}$ and the minimum. The error bars represent the standard deviations associated with the average.

To systematically study the dependence of the PMF well depth on N, we calculated the depth as a function of N from the PMFs in Figure 4A (see Figure 6). Here, the PMF well depth is defined as the average of the difference between the PMF value at $N_{1}=0$ (the left end point in Regime II, State B in Figure 5) and the minimum PMF (the middle point in Regime II, State E in Figure 5), as well as the difference between the PMF value at $N_{1}=N-N_{saturated\;CNT}$ (the right end point in Regime II, State H in Figure 5) and the minimum PMF. Note again that $N_{saturated\;CNT}$ is the number of molecules within the CNT when the CNT is filled with the molecules, and it is approximately 11~12. It is also worth noting that, in this calculation, we may consider the two end points of the entire PMF, including Regimes I and III, but since the transport processes in different regimes have different physical meanings, as discussed before, we did not use the values from different regimes. In Figure 6, we see that the PMF well depth increases with N, and interestingly, it increases in a quadratic way.

### 3.3. Potential Energy

**Figure 7:**
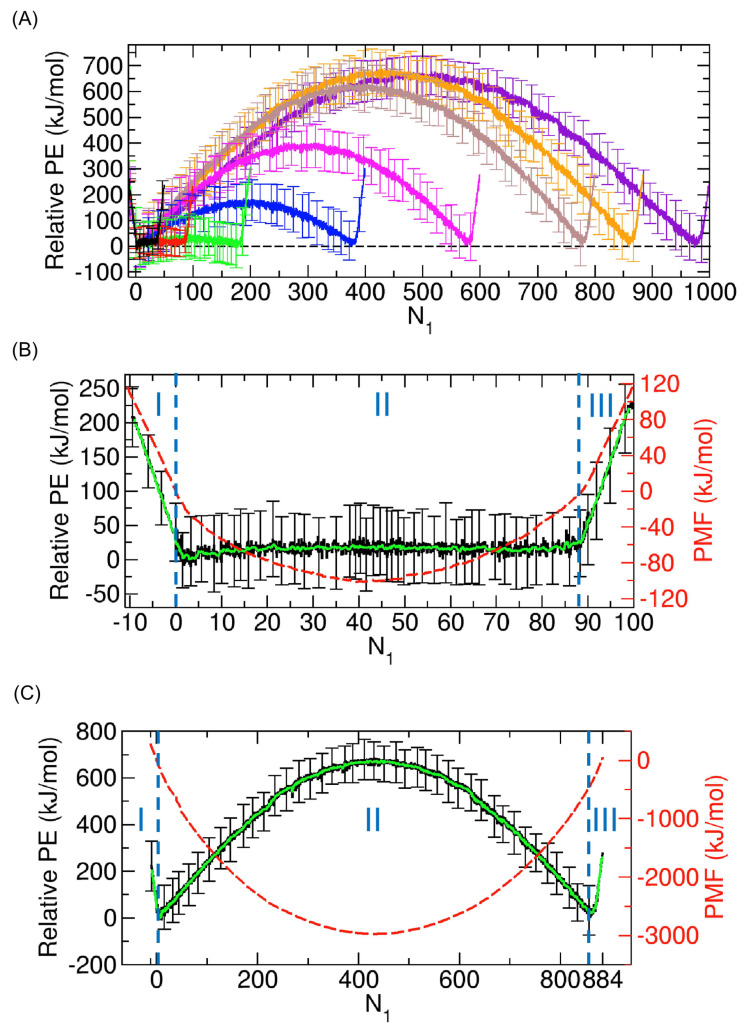
(**A**) Relative potential energies (PEs) for systems of 50, 100, 200, 400, 600, 800, 884, and 1000 nonpolar molecules as functions of $N_{1}$. We used the same color scheme as in Figure 4A to represent the cases with varying numbers of molecules. Here, the relative PE was obtained by making the minimum of the running-averaged values over 10 data points zero. The error bars indicate the standard deviations. (**B**) Relative PE (black) for the system of 100 molecules, along with the PMF (red) in Figure 5A. The running-averaged values over 10 data points are shown in green. (**C**) The same plots as those in (**B**) but with 884 molecules and with the PMF in Figure 5B.

To better understand the thermodynamic changes associated with the PMFs, we calculated the potential energy changes as functions of $N_{1}$, and we plotted them in a way such that the minima of the 10-point running averages are set to zero. The result is shown in Figure 7A. In contrast to the PMF change in Figure 4A, where a well is observed, a potential barrier is present in Regime II. To obtain deeper insight, we further investigated the two specific cases of N=100 and N=884 again in detail, which are shown in Figure 7B and Figure 7C, respectively.

In the case of 100 molecules in Figure 7B, we observe that the changes in the relative potential energy (black) and the PMF (red) are strongly correlated in both Regimes I and III. This correlation suggests that the changes in the PMF within these regimes could be primarily driven by the corresponding potential energy changes. These trends in Regimes I and III also appear in the case of water molecules [8]. Therefore, we can similarly interpret that, as more molecules enter the CNT inner space, the potential energy decreases since the molecules can considerably interact with the CNT and the state becomes thermodynamically more stable. This implies that the potential energy change due to the interaction between the molecules and the CNT is dominant, which will be further discussed later. Likewise, the observations in Figure 7C for 884 molecules exhibit analogous trends in Regimes I and III to those described above for 100 molecules.

However, in Regime II, the changes in the relative potential energy and the PMF do not display any noticeable correlation in either case (see Figure 7B,C); for 884 molecules, they are actually opposite. This suggests that the relative potential energy change is not the primary driving force for the PMF change; rather, another factor is at play, which will be discussed later. As previously discussed, the PMF trend in Regime II differs from that for water molecules (see Figure 4B), but the trend in the relative potential energy in Regime II is similar to that for water molecules in that both have a barrier. As explained in our prior work [8], the barrier arises due to the interaction between the transported molecules. Note that, in the previous case with water, an additional electrostatic interaction exists, while in this case, the only nonbonded interaction is the LJ interaction. Specifically, when we consider the transition from State B (or State H) in Figure 5A,B to State E, a group of molecules in Compartment 2 (or Compartment 1) divides into two separate smaller groups in Compartments 1 and 2, respectively. This division reduces the interaction between molecules, leading to an increase in potential energy. Therefore, in Regime II, we expect that the interaction between molecules is the dominant factor driving the potential change.

**Figure 8:**
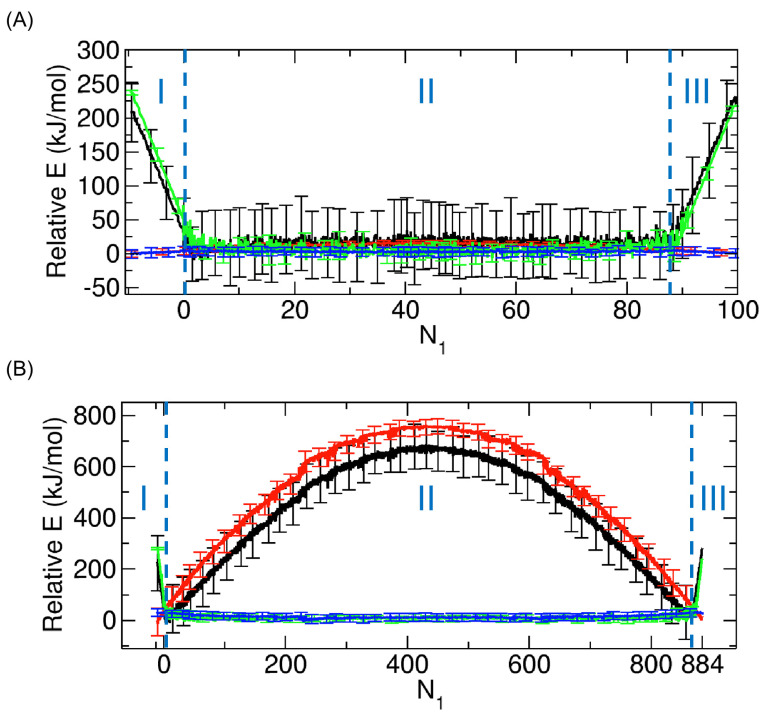
Decomposition of the relative potential energy (black) in Figure 7B,C into three components corresponding to the interactions between the molecules themselves (red), between the molecules and the CNT (green), and between the molecules and the compartments (blue) for (**A**) 100 and (**B**) 884 nonpolar molecules. Relative energies are calculated by setting the minima of the 10-point running averages to zero. The error bars indicate the standard deviations.

Expanding upon the prior discussion, to quantitatively ascertain the dominant interaction that drives the change in the relative potential energy, we decomposed the change into the contributions from interactions between the molecules and the CNT, between the molecules themselves, and between the molecules and the compartments. We display this decomposition in Figure 8.

The calculation confirms that the interaction between the CNT and the molecules is dominant in the change in relative potential energy in Regimes I and III and that the interaction between the molecules themselves is dominant in Regime II. However, the potential due to the interaction between the molecules and Compartments seems to be independent of $N_{1}$, which suggests that the interaction does not play a role during the entire transport process.

Based on Figure 7 and Figure 8, this analysis of potential energy concludes that the relative potential energy profile exhibits a barrier in the middle, located at the state where half of the molecules are transported. This observation implies that achieving a state where the transport of molecules is halfway is not thermodynamically possible if potential energy governs the thermodynamics of transport and the barrier is high. However, in contrast, the PMF profile shows a deep well around the middle (see Figure 4A and Figure 5), suggesting that factors other than potential energy may come into play.

### 3.4. Energetic and Entropic Contributions to the Formation of the PMF Well

To identify the influences on the PMF beyond potential energy, we first investigated the relation between the changes in PMF and potential energy. In our NVT ensemble, the PMF represents the Helmholtz free energy change (∆H) due to molecular transport, and this ∆H is decomposed into the internal energy change (∆U) and the entropic change (−T∆S) [22]. Given the constant temperature T in our sampling simulations, the kinetic contribution (∆KE) to ∆U is practically zero. Thus, the only remaining contribution to ∆U is due to the potential energy change (∆PE). Consequently, ∆H=∆U−T∆S≅∆PE−T∆S. From this relation, we estimated the entropic change (−T∆S).

**Figure 9:**
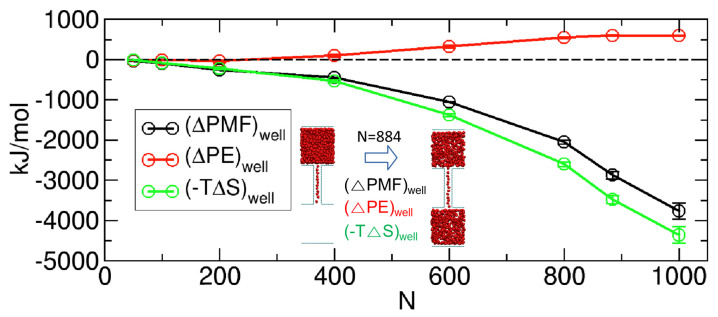
Energetic (${\left(∆PE\right)}_{well}$) and entropic (${\left(-T∆S\right)}_{well}$) contributions to the PMF change (${\left(∆PMF\right)}_{well}$) from the end states to the middle state in Regime II (from State B or from State H to State E in Figure 5) as functions of the total number of molecules N. The inset graphically depicts these changes for N=884. The error bars are the standard deviations of the two depths (e.g., the depth from State B in Figure 5 to State E in Figure 5 and the depth from State H to State E).

Considering that the energetic contribution (∆PE) tends to create a barrier, as discussed in Figure 7, we expect that the entropic contribution (−T∆S) is more inclined toward forming a well than a barrier to give the overall PMF a well-like profile. To verify this expectation, we calculated the entropic contribution to the formation of the PMF well. Importantly, the formation of the PMF well here refers to the change in free energy from the end state of Regime II (State B or State H in Figure 5) to the middle state of Regime II (State E in Figure 5), which is the negative value of well depth in Figure 6. The calculation for −T∆S was possible with −T∆S≅∆H−∆PE because the PMF (Figure 4A) and potential energy profiles (Figure 7A) were already calculated, allowing us to estimate ∆H (or ∆PMF) and ∆PE. The entropic and energetic contributions to the formation of the PMF well are shown in Figure 9.

In Figure 9, it becomes evident that the negative entropic change contributes positively to the formation of the PMF well by deepening the well. Conversely, the positive energetic change yields a negative contribution, causing the well to become shallower. Nevertheless, owing to the dominance of the entropic contribution over the energetic contribution in all cases, a PMF well emerges instead of a barrier.

## 4. Conclusions

In this work, we studied the thermodynamics of the transport of nonpolar molecules between two identical compartments through a CNT. Specifically, we calculated the PMF as a function of the coordinate indicating the progress of the molecular transport. For all the cases with 50, 100, 200, 400, 600, 800, 884, and 1000 molecules, the PMF profiles show a free energy well around the middle of the profiles, at which the molecules are equally distributed into the two compartments. Maxima occur at the ends of the profiles, in which case all the molecules are in only one of the two compartments. From the PMF profiles, we see that the transport of roughly half of the molecules from the initial state, where all molecules reside in only one of the two compartments, into the other initially empty compartment is spontaneous, but the further transport of more than half of the molecules is not spontaneous.

The former spontaneous process physically consists of two subprocesses: the first subprocess involves the transport of molecules to fill the CNT. Once the filling is complete, the second subprocess begins and continues until the concentrations in the two compartments are equal. In fact, the thermodynamic tendency of this spontaneity is manifested as the well depth in the PMF. We found that the well depth increases quadratically with the number of molecules to be transported. Moreover, additional analysis of the transport thermodynamics reveals that entropy plays a pivotal role in driving this second subprocess. In contrast, in our previous work with water molecules [8], we demonstrated that this second subprocess does not spontaneously occur due to barriers present in the PMF profiles. In that study, we also discussed that these barriers emerge as a result of strong water–water interactions, which implies that the energetic contribution to the PMF change is dominant over the entropic contribution. The absence and presence of such strong interactions between the transported molecules accounts for the differences in the PMF profiles that appeared in this work with nonpolar molecules and in the previous work with water molecules. It is important to note again that we modeled the nonpolar molecules by simply removing electric charges from the water molecules, and this removal significantly weakens the strength of the interaction between the molecules.

While our primary focus has been on examining the general properties of the PMF for transporting weakly interacting molecules using the charge-removed water model, it would also be interesting to explore diverse properties by employing various molecular models, including real molecules. For instance, by using molecules with distinct molecular geometries characterized by different bond lengths and angles, we can gain valuable insights into how molecular geometry affects the PMF. One such example is to compare the PMFs of nonpolar linear molecules and bent molecules, whose geometric differences could significantly affect the entropic contribution to the PMF.

Finally, by comparing this work and the previous work [8], we conclude that, when the interactions between transported molecules are strong, the PMF profile exhibits a barrier; conversely, when they are weak, it forms a well. This suggests that, when the interaction strength is intermediate, the PMF profile may have a flat region where the PMF value remains constant, neither a well nor a barrier. In such a flat region, we would anticipate spontaneous and reversible transport between the two components, as the chemical potential associated with the flat PMF region is zero. Furthermore, if the flat region is substantial, we would expect transport to occur on a large scale, potentially leading to significant fluctuations in the number of molecules within the compartments. Further investigation on this subject would be a promising avenue for future research.

## Figures and Tables

**Table 1:** LJ parameters of carbon atoms in CNT and graphene plate.

	CNT	Graphene Plate
$\sigma_{C}$ (nm)	0.3400	0.3400
$\varepsilon_{C}$ (kJ/mol)	0.3598	0.03598

**Table 2:** Comparison of values between original and modified TIP3P force fields.

	Original Value	Value in This Work
$r_{OH}$ (nm)	0.09572	0.09572
$\theta_{HOH}$ (deg)	104.52	104.52
$q_{O}$ (*e* units)	−0.834	0
$q_{H}$ (*e* units)	0.417	0
$\sigma_{O}$ (nm)	0.31506	0.31506
$\varepsilon_{O}$ (kJ/mol)	0.6364	0.6364

## Data Availability

The data underlying this study are available within the article.
